# Volume electron microscopy reveals bacterial endosymbiosis within host mitochondria

**DOI:** 10.1038/s42003-026-10301-0

**Published:** 2026-05-21

**Authors:** Jiří Týč, Luise Robbertse, Audra Salburg, Tereza Šedivá, Tomáš Bílý, Veronika Urbanová, František Kitzberger, Michaela Svobodová, Daria Tashyreva, Samuel Livingston, Patrick J. Keeling, Marie Vancová, Julius Lukeš, Jan Perner

**Affiliations:** 1 Institute of Parasitology, Biology Centre, Czech Academy of Sciences, České Budějovice, Czech Republic; 2 Faculty of Science, University of South Bohemia, České Budějovice, Czech Republic; 3 Department of Botany, University of British Columbia, Vancouver, BC Canada; 4 Present Address: Institute of Evolutionary Biology, Faculty of Biology, University of Warsaw, Warsaw, Poland

**Keywords:** Mitochondria, Symbiosis

## Abstract

Bacterial endosymbionts can inhabit certain eukaryotic compartments, yet their direct associations with mitochondria have remained poorly understood. Using a multiscale volumetric electron microscopy (vEM) framework spanning whole-cell reconstructions to subnanometer-scale electron tomography, we investigated mitochondria of two phylogenetically distinct eukaryotes: the tick *Ixodes ricinus* and the marine protist *Diplonema japonicum*. We show that *Midichloria mitochondrii*, the maternally-transmitted symbiont of *I. ricinus*, penetrates into the intracristal space of host mitochondria, inducing ≥60-fold expansion of mitochondrial cristae, with individual cristae exceeding 1 µm in width. In *D. japonicum*, we observed staged endosymbiotic interactions with the host mitochondrion, including cases of complete engulfment by both mitochondrial membranes. To describe the phenomenon of bacterial residency within mitochondria or in tight association with mitochondria, we introduce the term mitobiosis. Together, these findings establish mitochondria as niches for endosymbiotic bacteria and highlight vEM as a powerful tool for uncovering hidden organelle-microbe interactions.

## Abstract (web summary)

Volume electron microscopy reveals mitobiosis, a bacterial endosymbiosis with mitochondria, ranging from weak associations to established bacterial residency within mitochondria of evolutionarily distant eukaryotic hosts.

## Introduction

Eukaryotic cells are intricately organised systems, composed of specialised organelles, dynamic membrane networks, and precisely regulated biochemical environments^1^. In some organisms, including arthropods and protists^2^, two species-rich and abundant groups of eukaryotes, bacterial endosymbionts add a layer to cellular complexity of eukaryotes through diverse associations with host subcellular compartments^3,4^. The ability of these bacterial endosymbionts to interconnect with host´s compartmentalised metabolism and signalling^5^ underscores their remarkable adaptability with direct implications for the hosting cell. While in most cases bacteria reside in the cytoplasm, they can also be found in components of the endomembrane system such as phagosomes^6–9^, in the nucleus^10–13^, or, exceptionally, in the chloroplast^14^. Moreover, hydrogen-scavenging bacteria frequently tightly associate with hydrogenosomes in amoebae^15^, ciliates^16^, and parabasalids^17^. The establishment of bacteria within eukaryotic cells represents a complex interplay between recognition, response, and tolerance^18,19^. Once established in, the organelles can then engage in metabolic exchanges with the symbionts.

Mitochondria, the hubs of metabolic capacity of a hosting cell, believed to have arisen around 1.5 billion years ago from an alphaproteobacterial endosymbiont^20^, endowed the host cell with efficient aerobic respiration^21^. Previous studies have indicated, however, that several eukaryotic intracellular parasites can breach the exclusive partnership between mitochondria and a hosting cell. *Toxoplasma gondii*^22^, *Plasmodium berghei*^23^, *Trypanosoma cruzi*^24^, and Microsporidia^25^, or prokaryotic pathogens, such as *Chlamydia* spp.^26^ evolved mechanisms to associate with host cell mitochondria and exploit their biochemical anabolic capacities. Conversely, mitochondria are not defenceless themselves and are increasingly recognised as important players in cellular defence, capable of recognising intracellular microbes and mounting innate immune signalling and responses^27^. Recent work further indicates that mitochondria can actively constrain intracellular microbes by restricting access to key metabolites^28^, underscoring that mitochondrion-microbe proximity can reflect host defence mechanisms. In opposite cases, intracellular pathogenic bacteria can be encapsulated by mitochondria of micro-zooplanktonic cells, resulting in host cell lethality^29^. An additional, mechanistically distinct precedent comes from mitoviruses, non-virion-forming RNA elements that replicate within the mitochondrial matrix of eukaryotic hosts, supporting the idea that mitochondria can constitute accessible intracellular niches^30,31^. Together, these observations suggest that mitochondrion-associated microbes represent a recurring feature of eukaryotic cell biology, warranting closer structural investigation across diverse host lineages. Among the limited number of known mitochondrion-associated bacterial symbioses, *I. ricinus* ticks and the *D. japonicum* protists offer two phylogenetically distant systems for comparative structural analysis.

Original ultrastructural studies first provided evidence of the association of endosymbiotic bacteria in arthropods^32,33^, and freshwater and marine protists^29,34,35^. Although these studies relied on thin-section transmission electron microscopy (TEM) and could not resolve membrane topology in three dimensions (3D), they indicate that intimate mitochondrial associations have arisen repeatedly in evolution. Volume electron microscopy (vEM) mitigates this limit and enables reconstruction of whole cells and organelles in 3D^36,37^, resolving spatial relationships that are often ambiguous in conventional EM. Recent applications of vEM demonstrated its capacity to visualise organelle architecture and microbe–host interfaces with nanometre precision in the free-living^38,39^ and parasitic^40–42^ protists, as well as large multicellular organisms^43,44^. vEM can also provide the insight into the interactions between the pathogenic bacteria and surrounding host tissues^45^. Notably, pioneering focused ion beam scanning EM (FIB-SEM) studies have already provided 3D reconstructions of *M. mitochondrii* and revealed its possible impact on mitochondrial network dynamics^46^.

We show here that mitochondria, among other eukaryotic organelles, can serve as distinct cellular micro-niches, with discrete subcellular environments that can be colonised by bacterial symbionts. Combining multiple vEM modalities, including serial block-face scanning electron microscopy (SBF-SEM), FIB-SEM, array tomography (AT), and high-resolution electron tomography (ET), this integrated approach resolves mitochondrial colonisation in unprecedented volumetric detail, from cell-wide mapping of symbiont distributions to sub-organelle remodelling of cristae, demonstrating the power of vEM to uncover hidden dimensions of intracellular symbiosis.

## Results

### *Midichloria mitochondrii* colonises mitochondria of tick oocytes, including the intracristal space

**Fig. 1:**
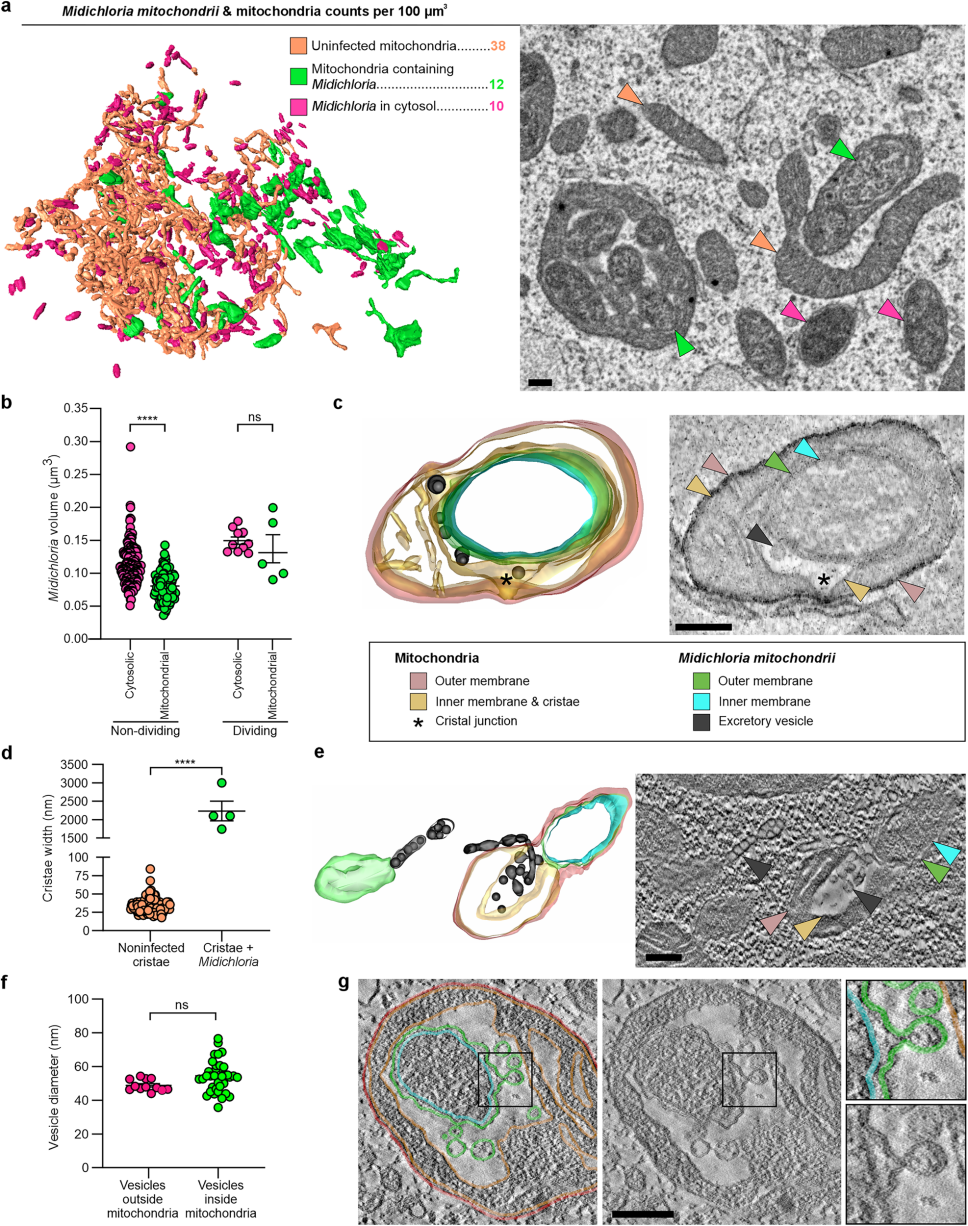
Intracellular distribution, size dynamics, and mitochondrial invasion of *Midichloria mitochondrii* in tick oocytes. **a** 3D model of part of a vitellogenic *I. ricinus* oocyte showing the spatial distribution of *M. mitochondrii* and host mitochondria (left) and a representative part of the AT section (right). Cytosolic bacteria are shown in magenta, mitochondria containing *M. mitochondrii* in green, and uninfected mitochondria in orange. **b** Quantification of bacterial volume (µm³) based on the AT data, comparing non-dividing and dividing *M. mitochondrii* in the cytosol and the mitochondrial compartments. Dividing cells are identified by furrow ingression. Mean volume for non-dividing cytosolic bacteria = 0.1129μm³ (s.e.m. = 0.0024) and for intra-mitochondrial = 0.0806μm³ (s.e.m. = 0.0021), *P* < 0.0001 (****). **c** Serial electron tomogram, with a total thickness of ~260 nm and a pixel size of 0.67 nm, provides evidence of intra-mitochondrial *M. mitochondrii* establishment within a crista, with a visible cristal junction (*). **d** Cristae width measurements taken on non-infected mitochondria (TEM) show a dramatic expansion after internalisation of *M. mitochondrii* (AT). Mean width of cristae in non-infected mitochondria = 32.87 nm (s.e.m. = 0.6; *n* = 200); mean width of cristae containing *M. mitochondrii* = 2,235 nm (s.e.m. = 268.7; *n* = 4). **e** ET reconstruction ( ~ 80 nm thickness, pixel size 0.98 nm) shows *M. mitochondrii* localised in the cytosol and within mitochondria. Chains of small, spherical vesicles appear continuous with the bacterial surface and are located both in the host cytoplasm and within the mitochondrion. Notably, some vesicles appear to traverse the mitochondrial membranes. **f** Diameter of membrane vesicles derived from *M. mitochondrii*. The mean diameter of vesicles (chains of small and spherical vesicles) derived from cytosolic bacteria (mean = 48.8 nm; s.e.m. = 0.978; *n* = 14) do not differ significantly from the mean diameter of vesicles derived from intra-mitochondrial bacteria (mean = 54.2 nm; s.e.m. = 1.471; *n* = 34). **g** ET image shows vesicle budding from the outer membrane of *M. mitochondrii*. Scale bars indicate 200 nm (**a**, **c**, **e**, **g**).

The tick *I. ricinus*, a predominant vector of horizontally-transmitted pathogenic microbes in Europe^47^, also hosts the vertically transmitted bacterial endosymbiont *M. mitochondrii*. This bacterium displays spatial partitioning within tick oocytes, with some cells occupying the cytosol while others inhabit the mitochondrion^48^. Here, we investigated its intracellular distribution and mitochondrial colonisation in *I. ricinus* oocytes using these two vEM techniques: array tomography (AT), and high-resolution electron tomography (ET), confirming the dual localisation of *M. mitochondrii* to the cytosol and mitochondria. A 3D ultrastructural reconstruction of a part of a *I. ricinus* oocyte revealed that the intra-mitochondrial localisation of *M. mitochondrii* is not rare, with 12 out of 50 mitochondria harbouring bacteria (Fig. 1a, Supplementary Video SV1).

Volumetric AT analysis showed that the non-dividing cytosolic *M. mitochondrii* were significantly larger (*P* < 0.0001) than their intra-mitochondrial counterparts (mean volumes: 0.1129 μm³ vs. 0.0806 μm³, respectively). Dividing bacteria, identified by characteristic furrow ingression, were larger than non-dividing bacteria in both compartments. Cytosol-dwelling dividing bacterial cells display a similar volume (mean volume: 0.1494 μm³, s.e.m.: 0.005481) compared to dividing bacteria in mitochondria (mean volume: 0.1372 μm³, s.e.m.: 0.02142 μm³ (Fig. 1b)). Serial ET captured details of mitochondrial residency by the endosymbionts (Fig. 1c), highlighting their localisation within the intracristal space, near the cristae junction. The presence of bacteria was associated with distortion and ballooning of the cristae membrane, indicating that a physical expansion of the occupied crista had occurred. The width of invaded cristae expanded dramatically, reaching up to 3 µm (mean: 2.24 µm; s.e.m.: 0.3 µm; *n* = 4), which is 68 times greater than the average width of uninfected cristae (mean: 32.9 nm, s.e.m.: 0.6, *n* = 200) (Fig. 1d). In oocytes of *I. ricinus*, we observed chains of small membrane vesicles produced by *M. mitochondrii* both in the cytosol and within the invaded mitochondria (Fig. 1e, f; Supplementary Fig. S1). Electron tomography suggests that the *M. mitochondrii*-derived vesicles may traverse mitochondrial membranes, as they extend from the intracristal space into the cytosol. Array tomography revealed that membrane vesicles can appear in substantial numbers (Supplementary Fig. S1). Multiple bacteria and membrane vesicles were found within the single intracristal space, possibly following bacterial division (Supplementary Fig. S2). The diameters of vesicles were then measured using ET models of *M. mitochondrii*. The mean diameter of vesicles produced by cytosolic bacteria, which form chains of small, spherical vesicles (single bacterium; Fig. 1e), was 48.83 nm (*n* = 14; s.e.m.: 0.98), and thus does not significantly differ from the mean diameter of vesicles derived from intra-mitochondrial bacteria (mean: 54.17 nm; *n* = 34; s.e.m.: 1.47) (Fig. 1f). Electron tomography indicates that these vesicles originate from the outer membrane of *M. mitochondrii*, while the bacterial inner membrane remains intact (Fig. 1g). Consistent with this origin, the vesicles appear to be bounded by a single lipid bilayer suggesting that they are outer membrane vesicles (OMVs).

### Endosymbionts of *Diplonema japonicum* span a range of associations with the host mitochondrion

**Fig. 2:**
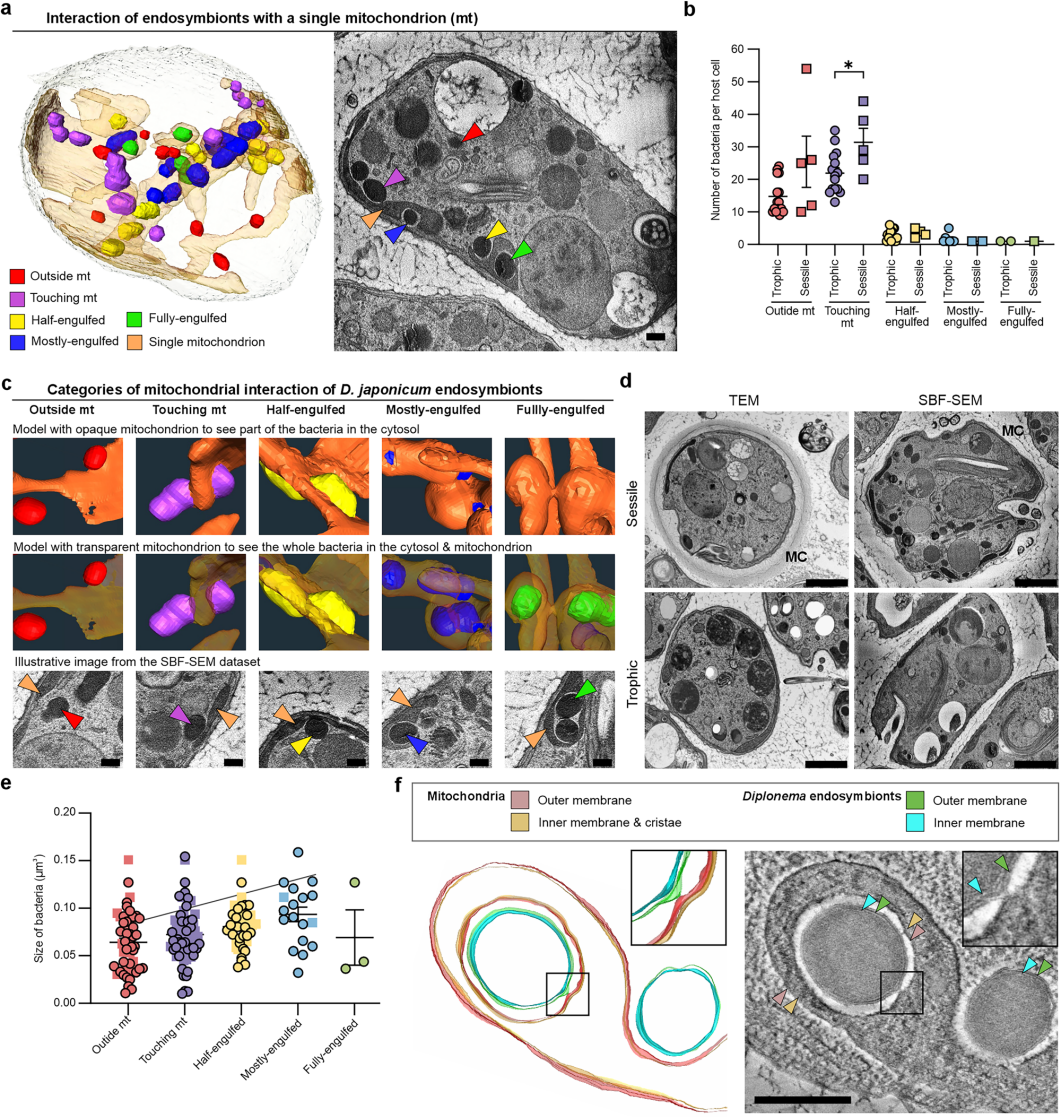
Spatial association between endosymbionts (*Nesciobacter abundans* and *Cytomitobacter primus*) with the *Diplonema japonicum* mitochondrion. **a** 3D model of a *D. japonicum* cell reconstructed from a SBF-SEM dataset (left) and a representative section from the same dataset (right). The single reticulated mitochondrion is shown in beige, and endosymbionts are colour-coded according to five manually assigned interaction categories with the mitochondrion. **b** Quantification of endosymbionts (*n* = 1086) across five categories of mitochondrial interaction in trophic (*n* = 15) and sessile cells (*n* = 5). **c** Shown are 3D models with an opaque mitochondrion and the corresponding transparent rendering, illustrating the relative positions of *D. japonicum* endosymbionts, as reconstructed from the SBF-SEM dataset of a trophic *D. japonicum* cell and its associated endosymbionts. Interaction categories are defined as follows: Outside mt: endosymbiont located outside the mitochondrion; Touching mt: endosymbiont in contact with the mitochondrial membrane ( < 50% of the bacterial surface in contact); Half-engulfed: endosymbiont partially enclosed by the mitochondrion ( ~ 50% engulfment); Mostly-engulfed endosymbiont partially enclosed by the mitochondrion ( ≥ 50% engulfment); and Fully-engulfed: endosymbiont completely enclosed within the mitochondrion with no contact with the cytosol. **d** *D. japonicum* trophic and sessile stages visualized by transmission electron microscopy (TEM) and SBF-SEM. The sessile stage of *D. japonicum* is rounded and surrounded by a definitive mucilaginous coat (MC) wrapped by long flagella around its body. See Supplementary Video SV3 for serial sections of this cell. While the trophic stage has short emergent flagella and lacks a mucilaginous coat. See Supplementary Video SV4 for serial sections of this cell. The scale bars indicate 2 µm. **e** Size distribution of endosymbionts in each interaction category (*n* = 129 bacteria) from six randomly selected trophic host cells, including both SBF-SEM (represented with circles, three cells) and FIB-SEM (represented with squares, three cells) datasets. Dot plot shows individual bacterial volumes (µm³). A significant positive correlation between bacterial size and extent of mitochondrial association was detected using a general linear model (*P* < 0.001). Bars represent mean counts, and error bars denote standard error. **f** Electron tomogram, with a total thickness of ~80 nm and a pixel size of 0.67 nm showing the membrane interface between the endosymbiont and the mitochondrial membranes. The inset highlights the site of close membrane apposition at high resolution. Scale bars indicate 200 nm (**a**, **c**, **d**, **f**).

*Diplonema japonicum* is a marine flagellate with morphology, ultrastructure, and lifestyle characteristic of this abundant group of heterotrophic protists^49^, hosting a single mitochondrion and two endosymbiont species, *Nesciobacter abundans* and *Cytomitobacter primus*. Initial observations from two-dimensional TEM of cultured cells revealed that the symbionts were closely associated with the host’s single, reticulated mitochondrion^49^. However, due to limitations in dimensionality, these data could not determine whether the bacteria were fully enclosed within the mitochondrion or only partially embedded in its structure. To address this, we used 3D reconstructions from SBF-SEM and FIB-SEM datasets (Supplementary Fig. S3), which revealed symbiont-mitochondrion associations to form a continuum, within which five recurring interaction categories could be distinguished (Fig. 2a; Supplementary Video SV2).

To capture this variation systematically, we classified symbiont-mitochondrion interactions into five categories based on increasing volumetric overlap between the two partners (Fig. 2a, c). To explore whether symbiont-mitochondrion proximity varies with host physiology, we quantified 1086 endosymbionts in 20 host cells, randomly sampled from the SBF-SEM dataset containing both sessile (*n* = 5) and trophic (*n* = 15) life cycle stages of *D. japonicum*, strain YPF1604 (Fig. 2b, d; Supplementary Videos SV3 and SV4). The majority of bacteria were either cytosolic (32.5%) or in close contact with the mitochondrial outer membrane over less than 50% of their surface area (47.6%). A substantial proportion (7.7%) was half or mostly enveloped by the mitochondrion, and in a small number of cases ( ~ 0.3%), the bacterium was fully enclosed, losing any direct connection with the surrounding host cytosol (Fig. 2b). To explore whether physical association with the mitochondrion correlated with symbiont size, we analysed bacterial volumes across interaction categories. Volumetric analysis revealed that endosymbiont size significantly correlated with the extent of mitochondrial association (*P* < 0.001), with larger bacteria having larger contact area with the mitochondrion (Fig. 2e). An exception to this pattern was the small subset of fully enveloped bacteria that did not follow this trend (mean: 0.06920 μm³, s.e.m.: 0.02908 μm³, *n* = 3) (Fig. 2e). A positive correlation between the symbiont’s size and proximity to the mitochondrion indicated interconnected biology of the intracellular bacterium and the organellar metabolism. Across TEM, SBF-SEM and FIB-SEM datasets, *D. japonicum* endosymbionts consistently exhibit an interface with the host mitochondrial membranes, with a thin layer of host cytosol remaining between them. Electron tomography provided higher-resolution of this interface, resolving a local region where the bacterial and mitochondrial membranes remain closely apposed while both membrane systems stay intact. Within this region, the tomogram revealed a local point of membrane contact, where the two membranes approach each other more closely than elsewhere along the interface (Fig. 2f).

## Discussion

The intracellular micro-niches of eukaryotic cells are available to bacterial endosymbionts, yet mitochondria have remained a poorly characterised site of symbiotic association. Volume electron microscopy reveals a bacterial endosymbiosis with mitochondria which we designate mitobiosis. It ranges from weak associations to established bacterial residency within mitochondria of evolutionarily distant eukaryotic hosts.

Specifically, we studied the capacity of *M. mitochondrii* to occupy the intercristal space and drive dramatic cristae remodelling in the mitochondria of *I. ricinus* oocytes. While our study is based only on a limited number of tick individuals, previous reports point to similar dramatic morphological changes associated with *M. mitochondrii* occupation of tick mitochondria^33,46,50^. Bacteria evolved a large array of adhesion molecules to eukaryotic cells to initiate their entrance^51^. Although many intracellular bacteria gain access to host cells by co-opting actin-dependent uptake pathways^52^, such mechanisms do not readily explain entry into mitochondria, which lack the actin-based cytoskeletal framework. Previous studies identified a possible set of proteins encoded in the genome of mitochondria-penetrating endosymbionts, that are likely to be decisive for the organellar entry^53^. These may interact with mitochondrial membrane proteins involved in membrane remodelling or fusion^54^, although the underlying bacterial factors remain unknown. Once established within the mitochondrion, *M. mitochondrii* localises between the folds of the inner membrane, including within regions where cristae architecture is preserved. In heavily colonised tick oocytes, individual mitochondria balloon up to accommodate multiple bacteria in their crista lumen, which swell dozens-fold in width. Quantitatively, the expansion of invaded cristae to micrometre-scale widths indicates that the inner-membrane architecture can be remodelled far beyond the range typically observed in eukaryotic cells^55^. The cristae are not just passive membrane folds, but in fact very specialised bioenergetic microdomains optimised for oxidative phosphorylation^56^, suggesting the mitochondrial metabolism needs to be severely compromised upon *M. mitchondrii* invasion. This deformation is consistent with earlier 3D work showing that *M. mitochondrii* infection is associated with altered fission-fusion dynamics^46^. Such extensive cristae remodelling is likely to alter local metabolite gradients, respiratory chain organisation, and mitochondrial performance in the infected mitochondria of *I. ricinus* oocytes. The *M. mitochondrii* endosymbionts in *I. ricinus* ticks, however, are known to be nearly 100% prevalent and maternally transmitted in ticks, with manifested loss of fitness in symbiont-free ticks, implying the *M. mitochondrii* symbionts confer a net advantage^57^. The molecular basis of the beneficial effect of harbouring *M. mitochondrii* has not yet been resolved.

In *D. japonicum*, the tight spatial association between the mitochondrion and the endosymbionts lacking all ATP-producing pathways^58^ suggests symbiont’s active import of ATP generated by the host. Since it is the larger endosymbionts that preferentially associate with the host’s single reticulated mitochondrion, it is plausible to assume that bacterial growth is energetically coupled to the mitochondrial output in the form of accessible ATP. However, since both endosymbionts cannot be distinguished based on ultrastructure alone, we could not determine whether both interact with the mitochondrion or if this association is specific to only one of them. Previous research indicated that these symbionts closely interacted with the mitochondrion^49^, but only our 3D reconstruction revealed that in this dynamic host-bacterial relationship the latter partner can be completely enveloped by the organelle losing a connection to the cytoplasm. Furthermore, the large SBF-SEM dataset allowed for acquiring quantitative data on the size and number of the endosymbionts and pinpointed the extent of their interaction with the mitochondrion. However, the nature of the interaction is distinct from that observed in *M. mitochondrii*. The symbiont did not cross the mitochondrial membranes but remained encapsulated within a compartment bounded by both mitochondrial membranes and containing remnants of the host cytosol. Interestingly, our ET also identified formation of direct points of contact between the endosymbiont and mitochondrion. Our electron tomography revealed points of direct membrane apposition, but not structured, protein-mediated “contact sites” *sensu stricto*. These localised contacts differ from the reproducible membrane spacing observed in other modalities and demonstrate that mitochondrial and bacterial membranes can come into immediate proximity. However, resolving whether specific tethering complexes mediate these interactions will require cryo-electron tomography. Interestingly, our volumetric analysis revealed a positive correlation between endosymbiont size and the extent of mitochondrial association (Fig. 2e). While increased bacterial size could reflect growth or preparation for division and thus higher energetic demand, this interpretation remains speculative and is not directly supported by our data. Alternatively, the observed size-dependent association may reflect differences in the physiological or metabolic state of the bacteria rather than cell division per se, which could influence their affinity for the mitochondrial environment. In addition, since the two endosymbionts cannot be distinguished ultrastructurally, the correlation may arise from preferential (or exclusive) mitochondrial association of one symbiont species that is intrinsically larger.

The intimate proximity of endosymbionts, particularly *M. mitochondrii*, and host mitochondria suggests the existence of a dedicated interface for molecular exchange. Early studies demonstrated that Gram-negative bacteria can export portions of their outer envelope as discrete lipid-bounded particles containing cell-wall and periplasmic components^59–61^. Subsequent studies showed that isolated bacterial membrane vesicles can physically associate with the outer membrane of other Gram-negative bacteria and undergo membrane fusion, resulting in incorporation of donor-derived envelope components into the recipient outer membrane and release of vesicle-associated material into the periplasm^62^. Whereas outer membrane vesicles were initially considered by-products of envelope instability or cell lysis, they are now recognised as regulated, selective products of the Gram-negative cell envelope generated during normal growth. Current models treat OMVs as integral components of bacterial physiology, functioning in envelope maintenance, cargo secretion, stress adaptation, and interactions with other cells and the environment^63^. Here, we show that *M. mitochondrii* produces vesicles of diameter around 50 nm. Vesicles of comparable size ( ~ 25–50 nm) were previously also recorded in *Chromulinavorax destructans*^29^ and symbionts of *D. japonicum*^64^. This observation suggests that endosymbiotic bacteria may also deploy the OMVs to interact with host cell mitochondria and/or other host cell components.

**Fig. 3:**
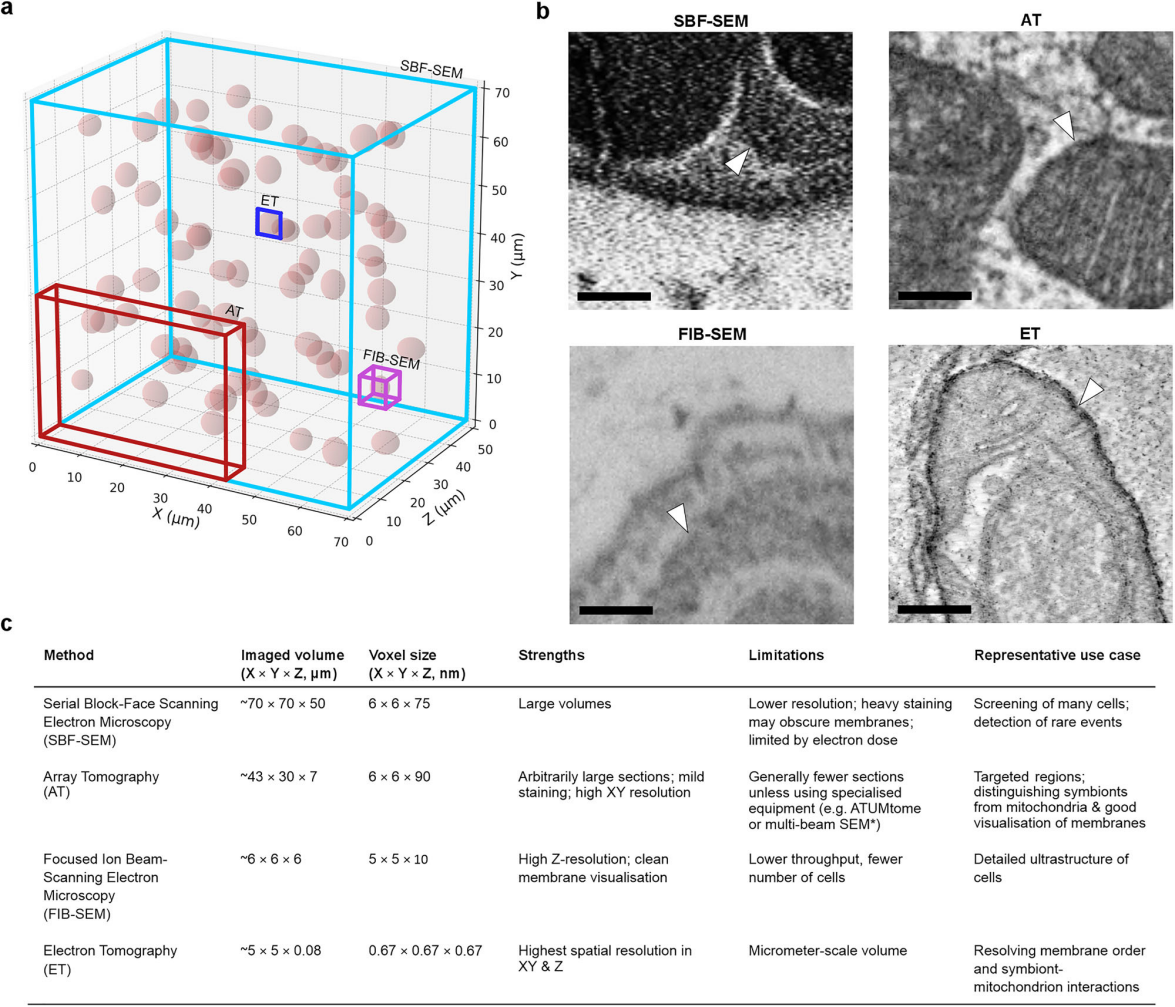
Complementary volumetric EM techniques applied to symbiont-mitochondrion interactions. **a** Schematic of multiple cells (balls) in space and illustration of imaging volumes (squares) for the different volume EM modalities: Serial Block-Face SEM (SBF-SEM, light blue), Array Tomography (AT, red), Focused Ion Beam SEM (FIB-SEM, magenta) and Electron Tomography (ET, dark blue). **b** Representative micrographs from each modality demonstrate the trade-off between volume coverage and resolution. SBF-SEM and FIB-SEM are derived from *D. japonicum*; the AT and ET are derived from *I. ricinus*. Arrowheads point towards mitochondria. Scale bars indicate 200 nm. **c** Summary table comparing the technical parameters of each vEM technique, including imaged volume, nominal voxel size, and methodological strengths and limitations. Together, these complementary approaches span scales from whole-cell symbiont distributions to ultrastructural details of the symbiont-mitochondrion membrane interface. * With specialised equipment, Array Tomography can image the largest volumes^72^.

Historically, studies of intracellular symbioses were largely constrained by 2D transmission electron microscopy^32–35^. While traditional ultrathin-section TEM provides exquisite ultrastructural detail, it captures only 2D projection images of thin sections and sacrifices the cell-wide spatial context needed to understand complex symbiont-organelle associations. Earlier ultrastructural reports across insects and other arthropods^32,33^, protists^34,35^, and additional lineages hinted that mitochondria do interact with bacteria in the cytoplasm, but the absence of volumetric continuity precluded unambiguous interpretation. Using continuous 3D reconstructions, we show that intimate mitochondrion-associated lifestyles occur in phylogenetically distant lineages and have likely evolved independently in unrelated hosts and endosymbiotic bacteria, consistent with the idea that mitochondria can constitute exploitable microenvironments under appropriate selective pressures. Our comparative analysis shows that “mitochondrion-associated bacteria” occupy distinct topologies: *M. mitochondrii* penetrates mitochondrial subcompartments, whereas *D. japonicum* symbionts retain remnants of the host cytosol, even when fully enclosed by mitochondria. The symbiont-mitochondrion interface posed distinct imaging challenges in diplonemids and ticks, requiring different volumetric EM strategies (Fig. 3, Supplementary Fig. S3). Earlier 3D reconstruction of *M. mitochondrii*, using FIB-SEM, revealed symbiosis-dependent morphological responses of tick mitochondria^46^. Our multiscale vEM approach (AT, SBF-SEM, FIB-SEM, and high-resolution ET) bridges whole-cell context and membrane-scale validation, revealing mitobiosis as a previously underappreciated dimension of intracellular symbiosis. In tick oocytes, by contrast, *M. mitochondrii* was abundant but morphologically difficult to distinguish from mitochondria in the SBF-SEM preparations, using more staining steps and agents. That is why array tomography was deployed to enable targeted sampling of parts of oocytes rich in symbiont-mitochondrion interfaces while preserving membrane contrast without additional contrast enhancement. This targeting also allowed us to restrict imaging to smaller but highly informative volumes, thereby saving acquisition time while still capturing the relevant interactions. Electron tomography then supplied the definitive ultrastructural resolution needed to unambiguously distinguish symbiont and mitochondrial membranes.

In diplonemids, symbionts engaged mitochondria in multiple ways, from peripheral contacts to complete enclosure within the organelle. To capture this diversity, SBF-SEM was used to screen large numbers of cells in bulk pellet samples; since examining many cells also crucial to detect rare intramitochondrial symbionts. FIB-SEM was applied for higher-resolution volumetric reconstructions and clearer visualisation of membranes, albeit at lower throughput. Finally, electron tomography resolved the number of membranes and identified points of contact. Thus, while the workflows differed, the rationale was unified: broad surveys for diverse contact forms, targeted enrichment for morphologically ambiguous cases, and electron tomography for ultrastructural validation. Taken together, these workflows establish a generalisable vEM strategy for analysing endosymbiont-organelle relationships across scales (Fig. 3; Supplementary Fig. S3). The datasets presented here extend this framework by examining two distantly related systems side by side, providing comparative insight into convergent structural adaptations for mitochondrial interaction.

Our results show that mitochondria are not only metabolic hubs for their host cell, but also habitats for intracellular symbionts. In *I. ricinus*, the fairly bulky ( ~ 400 nm in width) endosymbiont *M. mitochondrii* establishes itself awkwardly among the narrow mitochondrial cristae (20–50 nm), likely prompting extensive remodelling of the membrane architecture for its accommodation. In contrast, the *D. japonicum* endosymbionts illustrate a more fluid relationship, ranging from cytosolic proximity to rare, complete mitochondrial enclosure. Together, these systems capture two distinct examples of bacterium-mitochondrion associations. While our conclusions are grounded in membrane topology resolved in 3D, several constraints remain. For example, functional consequences of crista remodelling in *I. ricinus* are inferred rather than measured, which clearly necessitates next research steps. More broadly, our findings confirm that the mitochondrial interior is not an impermeable domain but can, in some cases, accommodate bacterial partners, implying that mitochondrial surveillance and symbiont tolerance can be reconciled under certain circumstances. The present work also provides a methodological framework for guiding future spatial characterisations of organelle-associated symbioses across diverse eukaryotes.

## Methods

### Biological material

The *I. ricinus* adult female ticks used in this study was obtained from the tick rearing facility of the Institute of Parasitology, Biology Centre, Czech Academy of Sciences (CAS). The colony was originally established from ticks collected by flagging in forested areas around České Budějovice, Czech Republic, and maintained in the laboratory for a maximum of two generations. Ticks were maintained under controlled laboratory conditions as previously described^65^. To complete the life cycle, unfed adult females were allowed to feed on guinea pigs until natural detachment. The tick ovaries were collected three days after spontaneous detachment from the host. Guinea pigs were bred and housed at the accredited animal facility of the Institute of Parasitology, Biology Centre CAS, in accordance with national regulations of the Ministry of Agriculture. Ticks originating from this region have previously been shown to harbour a stable, ovary-specific, monospecific microbiome of *M. mitochondrii*^66^. Laboratory animals were treated in accordance with the Animal Protection Law of the Czech Republic No. 246/1992 Sb., ethics approval No. 50-2022-P. The study was approved by the Institute of Parasitology, Biology Centre CAS and Central Committee for Animal Welfare, Czech Republic (Protocol No. 1/2015).

*Diplonema japonicum* used in this study was obtained from a laboratory culture maintained at the Institute of Parasitology, Biology Centre CAS. We used the same strain as described previously^49,58^. It was originally isolated from a marine sample collected in Japan and ever since was axenically cultured in seawater-based medium at temperatures between 15–20 °C. For long-term preservation, cultures have been kept in liquid nitrogen.

### Sample preparation

TEM samples of *D. japonicum* were processed as described elsewhere^49^. TEM samples of *I. ricinus* were processed as described previously^67^. Briefly, ovaries of *I. ricinus* ticks were dissected 3 days after full engorgement and spontaneous detachment of the host, fixed overnight at 4 °C in 2.5% glutaraldehyde, washed in 0.1 M phosphate buffer (pH 7.2) and incubated with 2% OsO_4_. Ovaries were dehydrated in an ascending acetone series, infiltrated with Epon812 resin and polymerised at 62 °C for 48 h. Ultrathin sections (70–90 nm) were placed on copper grids poststained with uranyl acetate for 30 min and lead citrate for 20 min and carbon-coated.

### Transmission electron microscopy

TEM images were acquired using a JEOL 1400 Flash microscope equipped with a SIS Xarosa CMOS camera. For cristae width measurements of *I. ricinus* mitochondria, a total of 200 cristae from 31 mitochondria, identified in five projection TEM images with a pixel size of 1.6 nm, were analysed in ImageJ. Measurements were performed by drawing a freehand line with a width of 20 pixels perpendicular to the cristae. A Plot Profile was generated, and the distance between the darkest points, representing the cristae membranes, was measured.

### Array tomography

For array tomography, an ovary from *I. ricinus* females was dissected and embedded as described above. Ultrathin sections (90 nm) were collected on a silicon wafer, which was glued to the SEM pins using double carbon tape, poststained with uranyl acetate for 45 min and lead citrate for 20 min, and carbon-coated. Array sections were imaged using Apreo SEM and MAPS software (Thermo Fisher Sci.). Serial images were acquired using OptiPlan mode: accelerating voltage 2.5 keV, probe current 0.2 nA, working distance 3.1 mm, pixel size 6 nm, and dwell time per pixel 10 µs. All quantitative measurements reported for *I. ricinus* originate from a single high-resolution array tomography dataset obtained from one laboratory-reared female tick. The analysed mitochondrial networks derive from a single oocyte and were randomly selected from this acquired volume.

### Electron tomography and serial sections electron tomography

All tilt series images for serial section electron tomography, of *D. japonicum* and *I. ricinus*, were acquired automatically using the SerialEM software package^68^ over a range of ±60° with a 1° tilt step. The tomogram in Fig. 1c was reconstructed from three serial tomograms acquired using a JEOL 2100 F equipped with a Gatan K2 Summit direct electron detector. The tomograms in Figs. 1e and 2f were acquired using the JEOL 1400 Flash. The tomogram in Supplementary Fig. S2 was reconstructed from two serial tomograms acquired using the JEOL 1400 Flash.

### Focused ion beam scanning electron microscopy

Leica ICE inside gold-coated copper HPF planchets (Technotrade Inc.) was used for high-pressure-frozen (HPF) cells of *D. japonicum* cells and stored in liquid nitrogen. Frozen samples were solvent-substituted under cryogenic conditions in an automatic freeze-substitution machine (Leica AFS2) with 2% uranyl acetate in anhydrous acetone for 4 days with the following schedule: −90 °C for 56 h, ramp to −50 °C over 8 h, stay at −50 °C for 8 h, ramp to −20 °C over 8 h, stay at −20 °C for 8 h, ramp to 4 °C over 8 h. Freeze-substitution medium was rinsed immediately upon removal from AFS with pre-cooled anhydrous acetone 3 times, then infiltrated with an ascending series of acetone:Epon812 at room temperature (minimum 2 h per step; 10%, 20%, 40%, 60%, 80%, and 3× consecutive passages in 100% resin). Samples were polymerised at 60 °C for 48 h. Resin-embedded blocks were trimmed and sectioned on an ultramicrotome (Leica UC7) to reveal an ROI containing well-preserved *D. japonicum* cells. The blocks were mounted onto an aluminium SEM stub with colloidal silver paste, then sputter-coated (Cressington 810HR) with platinum to a thickness of 50 nm. Imaging was done on Zeiss Crossbeam 350 using gallium ions for milling with three acquisition runs using slightly different voxel sizes in nm: (a) 5×5×10, (b) 5×5×20 (Supplementary Video SV5), and (c) 4×4×25.

### Serial block-face scanning electron microscopy (SBF-SEM)

Samples of *D. japonicum* were processed for high-pressure freezing as described elsewhere^38^, with the addition of 1% tannic acid staining and one more 2% OsO_4_ in 100% acetone after the initial freeze substitution mix containing 2% OsO_4_ and before the thiocarbohydrazide staining step. Resin-embedded blocks were trimmed and imaged using VolumeScope (Thermo Fisher Sci.). Serial images were acquired at 3.77 keV, 50 pA, 30 Pa with a resolution of 6 nm and 75 nm slice thickness, and dwell time per pixel of 3 µs. Quantitative analysis of symbiont-mitochondrion interactions was based on 1086 endosymbionts (*n* = 1086) identified across 20 host cells (*n* = 20; 15 trophic and 5 sessile) from a single SBF-SEM dataset. For analysis of the relationship between bacterial size and mitochondrial association, 129 individual endosymbionts (*n* = 129) were measured from six host cells (*n* = 6; three SBF-SEM and three FIB-SEM datasets).

### Data processing and segmentation

The vEM data were processed in combination of the most recent versions of FIJI^69^, MAPS (Thermo Fisher Sci.), Microscopy Image Browser (MIB)^70^, IMOD^71^, and Amira (Thermo Fisher Sci.). In MIB the segmentation was performed semi-automatically using the interactive 3D mode of SAM 2.1 (0.9 Gb backbone network) (arXiv:2408.00714) and manual tools for polishing the selections. The models were then rendered in Amira as a surface generated from the volume. The width of cristae was calculated from 5 individual measurements on 4 cristae in 4 mitochondria. A free-hand line, perpendicular to the cristae length, was drawn and measured. Tomogram reconstruction, the joining of serial tomograms, filtering with a nonlinear anisotropic diffusion filter, and model creation were performed using the IMOD software package^71^.

### Statistics and reproducibility

All statistical analyses were performed using GraphPad Prism version 10.4.1 (GraphPad Software, CA, USA). Data were first assessed for normality using the Shapiro–Wilk test. For comparisons between two groups, unpaired *t*-tests were used for normally distributed (parametric) data, while the Mann–Whitney *U* test was applied for non-parametric data. Statistical significance was defined as follows: *P* < 0.05 (*), *P* < 0.01 (**), *P* < 0.001 (***), and *P* < 0.0001 (****). The relationship between the bacterial volume and relative position of bacteria in or outside the mitochondria was tested using general linear models. The relative position was treated as a continuous variable so the statistical significance of the slope of the linear relationship was analysed at probability level 0.05. Statistical analysis was conducted in statistical programme R (version 4.5.1, R Core Team, 2025).

## Supplementary information

Supplementary information

Description of Additional Supplementary Files

Supplementary Data 1

Supplementary Data 2

Supplementary Data 3

Supplementary Data 4

Supplementary Data 5

Supplementary Video SV1

Supplementary Video SV2

Supplementary Video SV3

Supplementary Video SV4

Supplementary Video SV5

## Data Availability

The datasets supporting the figures in this study have been deposited in the BioImage Archive under accession number S-BIAD2413. Raw measurement data underlying the graphs are provided in the Supporting Data file.
