# Supplementary material for: Volume electron microscopy reveals bacterial endosymbiosis within host mitochondria
Source: Commun Biol. 2026 May 21;9:1086. doi: 10.1038/s42003-026-10301-0 (PMC13462478; doi:10.1038/s42003-026-10301-0)
Supplement: Supplementary file 1 [file 42003_2026_10301_MOESM6_ESM.pdf]

Trophic cells	b free in cy	b touching mt	half of b surrounded by mt	b surrounded by mt with opening	b fully surrounded by mt	total b count
Cell 3	24	35	5	1	0	65
Cell 1	23	16	5	0	0	44
Cell 2	13	23	4	0	0	40
Cell 4	13	22	3	5	0	43
Cell 5	16	13	2	1	0	32
Cell 6	22	21	2	0	0	45
Cell 7	11	20	1	0	0	32
Cell 8	10	32	3	1	0	46
Cell 9	11	28	1	0	0	40
Cell 10	16	17	1	2	0	36
Cell 11	11	27	2	0	0	40
Cell 12	9	17	4	0	1	31
Cell 13	22	17	6	1	1	47
Cell 14	10	25	2	1	0	38
Cell 15	10	16	1	0	0	27

b=bacterium

Sessile cells	b free in cy	b touching mt	half of b surrounded by mt	b surrounded by mt with opening	b fully surrounded by mt	total b count
Cell 1	26	20	4	0	0	50
Cell 2	54	44	3	1	0	102
Cell 3	12	29	2	0	0	43
Cell 4	25	38	5	1	1	70
Cell 5	10	26	0	0	0	36
