# Supplementary material for: Volume electron microscopy reveals bacterial endosymbiosis within host mitochondria
Source: Commun Biol. 2026 May 21;9:1086. doi: 10.1038/s42003-026-10301-0 (PMC13462478; doi:10.1038/s42003-026-10301-0)
Supplement: Supplementary file 2 [file 42003_2026_10301_MOESM7_ESM.pdf]

ALL	volume	category
SBFSEM 1	0,1272	5
	0,1207	4
	0,1111	4
	0,0975	4
	0,0927	2
	0,0907	4
	0,0869	2
	0,0802	3
	0,0769	3
	0,0724	3
	0,0673	2
	0,0634	3
	0,06	4
	0,0573	3
	0,0549	3
	0,051	4
	0,0475	3
	0,0449	3
	0,0439	5
	0,0417	1
	0,0405	3
	0,039	1
	0,038	3
	0,0365	5
	0,0333	1
	0,0327	1
	0,0321	4
	0,0297	1
	0,0272	1
	0,0251	1
	0,0178	1
	0,0148	1
	0,0127	2
	0,0124	2
	0,0108	1
	0,0101	2
SBFSEM 2	0,15433	2
	0,11657	2
	0,11342	4
	0,10339	3
	0,10339	3
	0,10216	3
	0,093396	3
	0,091922	1
	0,091417	1

categories interaction:

1	free
2	touching mt
3	half engulfed
4	mostly engulfed
5	fully engulfed by mt

	0,090995	1
	0,089324	1
	0,087839	4
	0,086036	2
	0,085847	1
	0,085539	2
	0,083265	4
	0,080158	2
	0,080125	1
	0,075986	2
	0,075454	1
	0,072706	1
	0,070106	1
	0,068375	3
	0,065856	4
	0,064233	2
	0,05953	1
	0,058031	2
	0,05765	2
	0,056819	2
	0,05653	2
	0,056047	1
	0,054977	4
	0,050282	2
	0,049488	2
	0,043421	1
	0,036796	1
	0,028985	2
	0,028812	2
SBFSEM 3	0,15894	4
	0,1282	4
	0,11289	2
	0,11071	2
	0,10591	1
	0,10392	4
	0,10231	2
	0,10163	1
	0,099441	3
	0,097227	1
	0,092721	3
	0,090145	3
	0,087863	2
	0,084783	1
	0,082471	3
	0,07992	3
	0,074628	2
	0,074128	3
	0,07334	1

	0,071995	1
	0,070999	3
	0,070959	3
	0,065831	2
	0,064884	1
	0,064282	2
	0,063112	2
	0,062586	2
	0,059322	1
	0,058741	2
	0,058344	2
	0,056676	1
	0,036242	2
	0,036083	1
	0,033291	2
FIB 1	0,11144	1
	0,091107	1
	0,084812	4
	0,08481	2
	0,084738	1
	0,084372	3
	0,082586	3
	0,082187	3
	0,080974	2
	0,077743	2
	0,074762	1
	0,070818	2
	0,068211	2
	0,064962	3
	0,054152	1
	0,05328	1
	0,049285	2
	0,048777	2
	0,045175	2
	0,043893	1
	0,03855	1
	0,033906	1
	0,031883	1
	0,030467	1
	0,029376	2
FIB 2	0,093796	2
	0,091011	3
	0,087311	2
	0,083482	3
	0,082399	2
	0,076026	2
	0,075207	2
	0,074373	2

	0,070119	2
	0,068154	3
	0,065606	1
	0,062652	1
	0,059717	2
	0,0589	3
	0,056761	3
	0,055649	2
	0,046233	2
	0,040931	1
FIB 3	0,1509	1
	0,094865	1
	0,11149	2
	0,10793	2
	0,10294	3
	0,097103	2
	0,092683	3
	0,088913	1
	0,08789	2
	0,087857	2
	0,086667	2
	0,084743	3
	0,074855	2
	0,065898	2
	0,064682	2
	0,054421	2
