# Supplementary material for: Volume electron microscopy reveals bacterial endosymbiosis within host mitochondria
Source: Commun Biol. 2026 May 21;9:1086. doi: 10.1038/s42003-026-10301-0 (PMC13462478; doi:10.1038/s42003-026-10301-0)
Supplement: Supplementary file 3 [file 42003_2026_10301_MOESM1_ESM.docx]

**Supplementary information**

**Supplementary Figure S1:** **AT images of densely packed *Midichloria*-containing mitochondria.** The images are taken from subsequent sections of the same region, highlighting the presence of the outer membrane vesicles (OMV)-like structures (indicated by yellow arrowheads).

**Supplementary Figure S2:** **sET (thickness ~140 nm, pixel size 0.66 nm) showing intra-mitochondrial stages, with multiple symbionts occupying the intracristal space.** Note the width of cristae exceeding 1 µm.

**Supplementary Figure S3: Comparison of different EM imaging methods used to examine mitobiosis. a)** Overview of AT section containing multiple oocytes; Horizontal Field of View (HFW) = 0.5 mm, best candidate ROIs are indicated in green and yellow. **b)** Section from the SBF-SEM diplonemid dataset illustrating how many cells can be imaged together; HFW = 70 µm. **c)** Example of the FIB-SEM dataset showing cross-section through one of the three cells imaged; HFW = 6 µm.

**Supplementary Video SV1: Part of the AT dataset and a 3D model of vitellogenic *I. ricinus* oocyte showing the spatial distribution of *M. mitochondrii* and host mitochondria.** The mitochondria are shown in beige, and intra-mitochondrial *M. mitochondria* cells are shown in colours.

**Supplementary Video SV2: Part of the SBF-SEM dataset and a 3D model of a *D. japonicum* cell.** The reticulated mitochondrion is shown in beige, and endosymbionts are colour-coded according to five interaction categories with the mitochondrion: red, outside mt; violet, touching mt; yellow, half-engulfed; blue, mostly-engulfed; green, fully-engulfed.

**Supplementary Video SV3:** Part of the SBF-SEM dataset showing serial sections through a sessile *Diplonema japonicum* cell.

**Supplementary Video SV4:** Part of the SBF-SEM dataset showing serial sections through a trophic *Diplonema japonicum* cell.

**Supplementary Video SV5:** Representative FIB-SEM dataset showing a *Diplonema japonicum* cell.

**Supplementary Data File**. Raw data underlying all graphs shown in the figures. The Excel file contains the raw data used to generate all graphs presented in the main figures. Each graph is provided in a separate worksheet.
