# Supplementary material for: Volume electron microscopy reveals bacterial endosymbiosis within host mitochondria
Source: Commun Biol. 2026 May 21;9:1086. doi: 10.1038/s42003-026-10301-0 (PMC13462478; doi:10.1038/s42003-026-10301-0)
Supplement: Supplementary file 4 [file 42003_2026_10301_MOESM2_ESM.docx]

**Description of Additional Supplementary Files**

**Supplementary Data 1-5:** Raw data underlying all graphs shown in the figures. The Excel file contains the raw data used to generate all graphs presented in the main figures. Each graph is provided in a separate worksheet.

**Supplementary Video SV1: Part of the AT dataset and a 3D model of vitellogenic *I. ricinus* oocyte showing the spatial distribution of *M. mitochondrii* and host mitochondria.** The mitochondria are shown in beige, and intra-mitochondrial *M. mitochondria* cells are shown in colours.

**Supplementary Video SV2: Part of the SBF-SEM dataset and a 3D model of a *D. japonicum* cell.** The reticulated mitochondrion is shown in beige, and endosymbionts are colour-coded according to five interaction categories with the mitochondrion: red, outside mt; violet, touching mt; yellow, half-engulfed; blue, mostly-engulfed; green, fully engulfed.

**Supplementary Video SV3:** Part of the SBF-SEM dataset showing serial sections through a sessile *Diplonema japonicum* cell.

**Supplementary Video SV4:** Part of the SBF-SEM dataset showing serial sections through a trophic *Diplonema japonicum* cell.

**Supplementary Video SV5:** Representative FIB-SEM dataset showing a *Diplonema japonicum* cell.
